# Electromyographic activity in deadlift exercise and its variants. A systematic review

**DOI:** 10.1371/journal.pone.0229507

**Published:** 2020-02-27

**Authors:** Isabel Martín-Fuentes, José M. Oliva-Lozano, José M. Muyor

**Affiliations:** 1 Health Research Centre, University of Almería, Almería, Spain; 2 Laboratory of Kinesiology, Biomechanics and Ergonomics (KIBIOMER Lab.), Research Central Services, University of Almería, Almería, Spain; University of Pittsburgh, UNITED STATES

## Abstract

The main purpose of this review was to systematically analyze the literature concerning studies which have investigated muscle activation when performing the *Deadlift* exercise and its variants. This study was conducted according to the Preferred Reporting Items for Systematic Reviews and Meta-Analysis Statement (PRISMA). Original studies from inception until March 2019 were sourced from four electronic databases including PubMed, OVID, Scopus and Web of Science. Inclusion criteria were as follows: (a) a cross-sectional or longitudinal study design; (b) evaluation of neuromuscular activation during *Deadlift* exercise or variants; (c) inclusion of healthy and trained participants, with no injury issues at least for six months before measurements; and (d) analyzed “sEMG amplitude”, “muscle activation” or “muscular activity” with surface electromyography (sEMG) devices. Major findings indicate that the biceps femoris is the most studied muscle, followed by gluteus maximus, vastus lateralis and erector spinae. Erector spinae and quadriceps muscles reported greater activation than gluteus maximus and biceps femoris muscles during *Deadlift* exercise and its variants. However, the *Romanian Deadlift* is associated with lower activation for erector spinae than for biceps femoris and semitendinosus. *Deadlift* also showed greater activation of the quadriceps muscles than the gluteus maximus and hamstring muscles. In general, semitendinosus muscle activation predominates over that of biceps femoris within hamstring muscles complex. In conclusion 1) Biceps femoris is the most evaluated muscle, followed by gluteus maximus, vastus lateralis and erector spinae during Deadlift exercises; 2) Erector spinae and quadriceps muscles are more activated than gluteus maximus and biceps femoris muscles within Deadlift exercises; 3) Within the hamstring muscles complex, semitendinosus elicits slightly greater muscle activation than biceps femoris during Deadlift exercises; and 4) A unified criterion upon methodology is necessary in order to report reliable outcomes when using surface electromyography recordings.

## Introduction

Resistance training provides several health benefits related to enhancing muscle strength, reversing muscle loss, reducing body fat, improving cardiovascular health, enhancing mental health and increasing bone mineral density [1–6]. Accordingly, resistance training should be considered essential for the whole population, but it is even more relevant when the target is the transference into some specific activity or daily life tasks [7, 8], injury prevention [9] or maximizing sports performance [10].

Free-weight resistance training is already well known as a key point in every strength training program [11–13]. In categories of creating diverse stimulus for muscle groups, different modalities such as barbell, kettlebells, hexagonal bars or dumbbells devices are typical recurring resources for coaches and trainers [14, 15]. Besides, other implements which can considerably modify the exercise load profile are elastic bands [2, 25], chains [29] or Fat Gripz devices [24].

It is essential to be acquainted with which muscles are activated during certain exercises and to compare different movement patterns when choosing exercises for a concrete objective [16]. Surface electromyography (sEMG) is one of the main tools used to measure muscle activation, and it can be defined as an electrophysiological recording technology used for the detection of the electric potential crossing muscle fiber membranes [17]. Thereby, task-specific data regarding motor unit recruitment patterns are reported through sEMG. For instances, athletes have the possibility to perform a concrete exercise when targeting a particular muscle [18, 19].

*Deadlift*, *Squat* and *Bench Press* are basic resistance exercises performed in several training programs for improving physical fitness in athletes [20]. This explains the great interest in studying muscle activation, which also translates these movements into some of the most investigated exercises in the current literature using sEMG [14, 21, 22]. *Deadlift* is frequently performed primarily when the goal is the strengthening of thigh and posterior chain muscles; specifically gluteus, hamstrings, erector spinae and quadriceps [23, 24]. Thus, *Deadlift* is classified as one of the most typical resistance exercise for posterior lower limb strengthening, as well as its variants [25]. Moreover, *Deadlift* has been mentioned in numerous studies comparing this exercise with other variants such as *Stiff Leg Deadlift* [26], *Hexagonal Bar Deadlift* [22] or *Romanian Deadlift* [27]. It has also been contrasted with other less popular variants such as *Sumo Deadlift* [13], unstable devices [28] and elastic bands *Deadlift* [8], among others.

To the best of our knowledge, there is no comprehensive review of the current literature concerning *Deadlift* movement pattern, and there is significant controversy when determining which muscles are involved within each *Deadlift* variants. For instance, the greatest muscle activation has been reported for the biceps femoris compared with the erector spinae and gluteus maximus during *Deadlift* [8], whereas Snyder et al. (2017) found greater erector spinae activation in comparison with gluteus maximus and biceps femoris. In contrast, Andersen et al. (2018) reported maximal activation for biceps femoris versus gluteus maximus and erector spinae for the same tested movement.

Thus, the main purpose of this manuscript was to systematically review the current literature investigating muscle activation measured with sEMG of muscles recruited when performing the *Deadlift* exercise and all its best-known variants. An increased understanding of the muscle activation that occur during these exercises will provide the researcher, clinician and athletes with relevant information about the use of the best exercise to activate a specific muscle or group of muscles associated with the *Deadlift* and its variants.

## Methods

This systematic review was reported and developed following the Preferred Reporting of Systematic Reviews and Meta-Analysis (PRISMA) guidelines [29, 30]. The protocol for this systematic review was registered on PROSPERO (CRD42019138026) and is available in full on the National Institute for Health Research (https://www.crd.york.ac.uk/prospero/display_record.php?ID=CRD42019138026). The quality of included studies was assessed by two reviewers using the PEDro quality scale, which consists on eleven questions and distributes the score proportionally to the total amount of questions included. However, due to the inability to blind researchers and trainees, three of eleven questions were excluded from the scale resulting in a maximum of eight [17].

A literature search of PubMed, OVID, Scopus & Web of Science electronic databases was performed from March–April 2019. Reviews included publications from inception until March 2019.

The search strategy conducted in the different databases, along with Medical Subject Heading (MeSH) descriptors, related terms and keywords used were as follows; (a) PubMed & OVID: (deadlift OR "dead-lift" OR "romanian deadlift" OR "stiff-leg deadlift" OR "barbell deadlift" OR "hexagonal bar deadlift" OR "hip hinge" OR "hip extension") AND ("resistance training" OR "strength training" OR "resistance exercise" OR "weight lifting" OR "weight bearing") AND ("muscular activity" OR "muscle activation" OR electromyography OR electromyographical OR electromyographic OR electromyogram OR "surface electromyography" OR semg OR EMG) (b) Scopus: (TITLE("deadlift" OR "dead-lift" OR "romanian deadlift" OR "stiff-leg deadlift" OR "barbell deadlift" OR "hexagonal bar deadlift" OR "hip hinge" OR "hip extension") AND ("resistance training" OR "strength training" OR "resistance exercise" OR "weight lifting" OR "weight bearing") AND ("muscular activity" OR "muscle activation" OR "electromyography" OR "electromyograpical" OR "electromyographic" OR "electromyogram" OR "surface electromyography" OR "sEMG" OR "EMG")); (c) Web of Science: ALL = (((deadlift* OR "dead-lift"* OR "romanian deadlift"* OR "stiff-leg deadlift"* OR "barbell deadlift"* OR "hexagonal bar deadlift"* OR "hip hinge"* OR "hip extension"*) AND ("resistance training"* OR "strength training"* OR "resistance exercise"* OR "weight lifting"* OR "weight bearing"*) AND ("muscular activity"* OR "muscle activation"* OR electromyograpical* OR electromyographic* OR electromyogram* OR "surface electromyography"* OR semg* OR EMG*))).

Studies were included if they met the following criteria:

- cross-sectional or longitudinal (experimental or cohorts) study design;
- evaluated neuromuscular activation during *Deadlift* exercise or variants;
- included healthy and trained participants, with no injuries for at least six months before measurements;
- analyzed “sEMG amplitude”, “muscle activation” or “muscular activity” with surface electromyography devices (sEMG);

Most articles found were written in English, but there were no language restrictions. Reviews, congress publications, theses, books, books chapters, abstracts, and studies with poor protocol description or insufficient data were not included. Studies whose participants did not have at least six months of resistance training experience were excluded. We also excluded all studies in which participants were under eighteen years old due to underdevelopment of strength and coordination [31]. Studies reporting muscle activation only from upper limbs during *Deadlift* exercise were also considered.

As different terms are related to the same concept, in categories of unifying criteria, the “muscle activation” term will be used when referring to “sEMG amplitude”, “muscle excitation”, “muscle activity”, “neuromuscular activity” or similar.

Articles were selected by two independent reviewers according to inclusion and exclusion criteria. After eliminating duplicates, the titles and abstracts were analyzed and if there was not enough information, the full text was evaluated. All studies identified from the database searches were downloaded into the software EndNote version X9 (Clarivate Analytics, New York, NY, USA).

**Fig 1:**
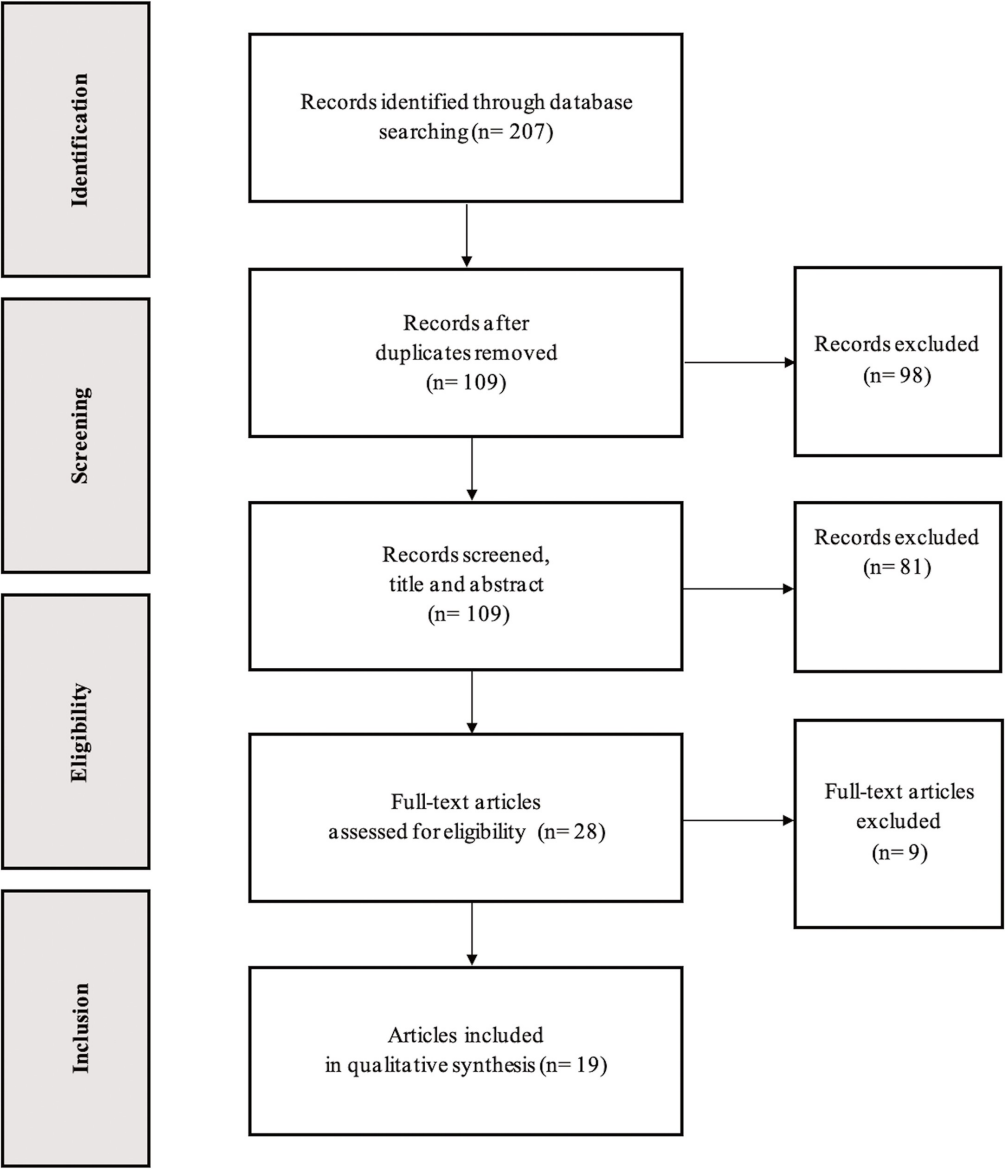
Flowchart.

Every decision was approved by both reviewers. However, a third reviewer was consulted in case of disagreement. The whole search process took two weeks. All steps taken are thoroughly described in the flow chart (Fig 1).

During the data extraction process, the following information was collected from every study: reference, exercise-movements measured, sample size (n), gender, age (years), experience (years), evaluated muscles, electrodes location, limb tested (non-dominant/dominant), sEMG collection method, sEMG normalization method, outcomes, percentage maximal voluntary isometric contraction (% MVIC), and main findings.

Muscle activation was the main data gathered, dividing eccentric and concentric sEMG activity data when reported. All studies finally selected reported muscle activation of every muscle and exercise separately. Furthermore, data related to exercise loading and exercise description details were collected.

Data collected in this review could not be analyzed as a meta-analysis since there was not enough homogeneity in terms of the type of analysis and methods carried out amongst studies. Therefore, a qualitative review of the results was conducted.

## Results

### Search results

A total of 207 articles were identified from an initial survey executed by two independent reviewers. 98 of these articles were duplicated, which led to a remaining amount of 109 in the process. The next step involved reading the title and abstract with the purpose of eliminating all those not meeting the inclusion criteria. Finally, twenty-eight articles were fully read, and nineteen of these were eventually selected for the review (Fig 1). The publication date of all selected articles ranged from 2002 to January 2019. Additionally, all studies were categorized as having a good/excellent quality in the methodological process based on the PEDro quality scale.

All selected articles presented a cross-sectional design. In fact, most experimental studies found used an untrained participant sample, so they were excluded. Regarding experience time, all participants had at least six months of previous resistance training experience, although some studies did not report the exact experience time of participants (Table 1).

**Table 1:** Data gathered from selected articles regarding intervention, sample size, gender, training experience, age, sEMG collection method, outcomes and main findings.

Reference	Exercises tested	Sample	Age (years)	Experience (years)	sEMG collection method	Activity sEMG recorded of muscles:	Main findings
Krings et al. (2019) [32]	Deadlift versus fat gripz deadlift	15 Men	22.4 ± 2.4	Not indicated	1 rep 1RM	Biceps brachialis, triceps braquialis and forearm muscles	Greater forearm activation and significant decrease in 1RM during fat gripz deadlift
Andersen et al. (2019) [8]	Deadlift versus FW-2EB and FW-4EB	15 Men	23.3 ± 2.2	3.9 ± 1.9	2 reps 2RM	Gluteus maximus, vastus lateralis, biceps femoris, semitendinosus and erector spinae	Greater erector spinae activation when more elastic bands added
McCurdy et al. (2018) [40]	Stiff leg deadlift versus back squat and modified single leg squat	18 Women	20.9 ± 1.1	1–5 years	3 rep with 8RM	Gluteus maximus and hamstrings	Greater gluteus maximus activation than hamstrings for all exercises. Modified single leg squat elicited the greatest activation
Lee et al. (2018) [27]	Deadlift versus Romanian deadlift	21 Men	22.4 ± 2.2	> 3 years	5 rep 70% of RD 1RM	Gluteus maximus, rectus femoris and biceps femoris	Greater gluteus maximus and rectus femoris activation for deadlift
Korak et al. (2018) [39]	Deadlift versus paralell back squat and paralell front squat	13 Women	22.8 ± 3.1	> 1 year	3 reps 75% 1RM	Gluteus maximus, biceps femoris, vastus medialis, vastus lateralis and rectus femoris	Greater gluteus maximus activation during front and back squat in comparison to deadlift
Edington et al. (2018) [35]	ISOMETRIC: close-bar deadlift versus far-bar deadlift	5 men & 5 women	32 ± 10	6.05 ± 3.35	3 trials in both starting positions. ISOpull	Gluteus maximus, biceps femoris, vastus lateralis, erector spinae and latissimus dorsi	Greater erector spinae and biceps femoris activation than the rest of muscles for both exercises. Greater vastus lateralis activation during far-bar deadlift
Andersen et al. (2018) [22]	Deadlift versus hexagonal bar deadlift and hip thrust	13 Men	21.9 ± 1.6	4.5 ± 1.9	1 rep 1RM	Gluteus maximus, biceps femoris and erector spinae	Greater biceps femoris activation during deadlift. Greater gluteus maximus activation during hip thrust. Erector spinae activation showed no differences among exercises
Snyder et al. (2017) [36]	Deadlift versus walk-in deadlift machine (2 different feet positions)	2 women & 13 men	18–24	Not indicated	3 reps 80% 3RM	Gluteus maximus, biceps femoris, vastus lateralis and erector spinae	Greater erector spinae activation during deadlift. Greater gluteus maximus and lower vastus lateralis activation during deadlift compared to walk-in machine deadlifts
Iversen et al. (2017) [41]	Stiff leg deadlift versus stiff leg deadlift with elastic bands	17 men & 12 women	25 ± 3 men 25 ± 2 women	Not indicated	3 reps 10RM	Gluteus maximus, biceps femoris, semitendinosus, vastus medialis, vastus lateralis, rectus femoris, erector spinae and external oblique	Greater activation for all muscles during conventional resistance exercises compared to elastic band deadlifts. Rectus femoris showed no differences activation among exercises
Bourne et al. (2017) [16]	Stiff leg deadlift versus unilateral stiff leg deadlift, hip hinge, 45° hip extension and nordic hamstring exercise	18/10 Men	23.9 ± 3.1	Not indicated	6 reps 12RM	Biceps femoris and semitendinosus	Greater semitendinosus concentric activation during unilateral stiff leg deadlift versus remaining exercises. Similar biceps femoris and semitendinosus activation during both deadlift exercises
Nijem et al. (2016) [37]	Deadlift versus deadlift with chains	13 Men	24.0 ± 2.1	Not indicated	3 reps 85% 1RM	Gluteus maximus, vastus lateralis and erector spinae	Greater gluteus maximus activation during deadlift. Greater erector spinae activation during the beginning of the movement for both exercises
Camara et al. (2016) [38]	Deadlift versus hexagonal bar deadlift	20 Men	23.3 ± 2.1	> 1 year	3 reps 65% 1RM & 3 reps 85% 1RM	Biceps femoris, vastus lateralis and erector spinae	Greater vastus lateralis activation; and lower biceps femoris and erector spinae activation during hexagonal bar deadlift
Schoenfeld et al. (2015) [42]	Stiff leg deadlift versus prone lying leg curl in machine	10 Men	23.5 ± 3.1	4.6 ± 2.2	1 set 8RM	Biceps femoris and semitendinosus	Greater upper biceps femoris and upper semitendinosus activation during stiff leg deadlift
McAllister et al. (2014) [44]	Romanian deadlift versus glute ham-raise, good morning and prone leg curl	12 Men	27.1 ± 7.7	8.6 ± 5.5	85% 1RM	Gluteus medius, biceps femoris, semitendinosus, erector spinae and medial gastrocnemius	Greater semitendinosus activation than biceps femoris and erector spinae activation for all exercises
Bezerra et al. (2013) [26]	Deadlift versus stiff leg deadlift	14 Men	26.7 ± 4.9	> 2 years	3 reps 70% 1RM	Biceps femoris, vastus lateralis, lumbar multifidus, anterior tibialis and medial gastrocnemius	Greater vastus lateralis activation during deadlift. Greater medial gastrocnemius activation during stiff leg deadlift
Chulvi-Medrano et al. (2010) [28]	Deadlift versus Bosu deadlift and T-Bow device deadlift	31	24.2 ± 0.4	> 1 year	Dinamic effort, 6 reps 70% of MVIC	Lumbar multifidus, thoracic multifidus, lumbar spinae and thoracic spinae	Greater overall activation during deadlift versus Bosu and T-Bow device deadlifts
Ebben (2009) [43]	Stiff leg deadlift versus unilateral stiff leg deadlift, good morning, seated leg curl, nordic hamstring exercise and squat	21 men & 13 women	20.3 ± 1.7	Not indicated	2 reps 6RM	Rectus femoris and hamstrings	Greater biceps femoris activation during seated leg curl and nordic hamstring than remaining exercises. Greater rectus femoris activation during squat
Hamlyn et al. (2007) [34]	Deadlift versus paralell squat	8 men & 8 women	24.1 ± 6.8	Not indicated	6 reps 80% 1RM	Lower abdominal, external oblique, lumbar-sacral erector spinae and upper lumbar erector spinae	Greater upper lumbar erector spinae activation during deadlift
Escamilla et al. (2002) [13]	Deadlift versus sumo deadlift (both with/without belt)	13 Men	20.1 ± 1.3	Not indicated	4 reps 12 RM	Gluteus maximus, biceps femoris, vastus medialis, vastus lateralis, rectus femoris, lateral and medial gastrocnemius, tibialis anterior, L3, T12, medial and upper trapezius, rectus abdominis and external oblique	Greater vastus medialis, vastus lateralis and tibialis anterior activation during sumo deadlift. Greater medialis gastrocnemius activation during deadlift. Greater rectus abdominis activation during belt deadlift and belt sumo deadlift

*Exercises abbreviations*: EB, elastic bands; FW, free weights.

*Other abbreviations*: ISOpull, isometric pulls; MVIC, maximal voluntary isometric contraction; reps, repetitions; RM, repetition maximum; ROM, range of motion.

No common criteria were followed when referring to the exercise loading at which exercises were evaluated during sEMG recordings. As a matter of fact, only two studies used a similar method, assessing one repetition maximum intensity (1RM) [22, 32]. Some studies measured a number of repetitions of xRM, whereas others measured a number of repetitions of a range between 65–85% of 1RM (Table 1), which could be considered in all cases as a submaximal load intensity [33].

Data regarding the studies’ general description and main findings are presented in Table 1, while Tables 2–5 contain data referring to muscle activation during *Deadlift* exercise and/or its variants. We found no unified criteria for the sEMG normalization method. Out of all included studies, seven reported data description regarding muscle activation in relation to exercise type and normalized sEMG activity as a percentage of maximal voluntary isometric contraction (% MVIC) (Table 2); three of them as percentage of peak root mean square (% peak RMS) (Table 3); two studies reported data expressed as absolute RMS values in microvolts (mV) (Table 4); and three studies expressed data as a percentage of 1 repetition maximum (% 1RM) (Table 5). In addition, there were four studies which were not included in the tables because they assessed the sEMG only from the upper limbs or showed the muscle activation in a different measurement unit than that used in our analysis [28, 32, 34, 35].

**Table 2:** Data description regarding sEMG activity in each study, in relation to exercise type and normalized sEMG activation expressed as mean or peak % MVIC.

Reference	Exercise	Gluteus Maximus	Biceps Femoris	Semitendinosus	Hamstrings	Vastus Lateralis	Vastus Medialis	Rectus Femoris	Erector Spinae
McCurdy et al. (2018) [40]	Stiff leg deadlift	51.1 ± 22.1% mean conc 29.9 ± 16.2% mean eccen	n/a	n/a	39.8 ± 16.6% mean conc 19.9 ± 11.3% mean eccen	n/a	n/a	n/a	n/a
Andersen et al. (2018) [22]	Deadlift	~95% mean	~108% mean	n/a	n/a	n/a	n/a	n/a	~86% mean
	Hexagonal bar deadlift (HBDL)	~88% mean	~83% mean	n/a	n/a	n/a	n/a	n/a	~82% mean
Iversen et al. (2017) [41]	Stiff leg deadlift	~42% peak conc ~17% peak eccent	~38% peak conc ~17% peak eccent	~44% peak conc ~22% peak eccent	n/a	~13% peak conc ~14% peak eccent	~10% peak conc ~9% peak eccent	~6% peak conc ~7% peak eccent	~69% peak conc ~38% peak eccent
	Stiff leg deadlift with elastic bands	~27% peak conc ~17% peak eccent	~20% peak conc ~17% peak eccent	~23% peak conc ~21% peak eccent	n/a	~12% peak conc ~14% peak eccent	~9% peak conc ~8% peak eccent	~5% peak conc ~6% peak eccent	~57% peak conc ~36% peak eccent
Bourne et al. (2017) [16]	Stiff leg deadlift	n/a	~55% mean conc ~23% mean eccen	~50% mean conc ~18% mean eccen	n/a	n/a	n/a	n/a	n/a
	Unilateral stiff leg deadlift	n/a	~50% mean conc ~26% mean eccen	~62% mean conc ~27% mean eccen	n/a	n/a	n/a	n/a	n/a
Schoenfeld et al. (2015) [42]	Stiff leg deadlift	n/a	~40% mean lower ~73% mean upper	~47% mean lower ~125% mean upper	n/a	n/a	n/a	n/a	n/a
Ebben (2009) [43]	Stiff leg deadlift	n/a	n/a	n/a	49±27% mean	n/a	n/a	n/a	n/a
	Unilateral stiff leg deadlift	n/a	n/a	n/a	48±39% mean	n/a	n/a	n/a	n/a
	Good morning	n/a	n/a	n/a	43±16% mean	n/a	n/a	n/a	n/a
Escamilla et al. (2002) [13]	Deadlift	35±27% mean	28±19% mean	27±23% mean	n/a	40±22% mean	36±25% mean	19±16% mean	n/a
	Sumo deadlift	37±28% mean	29±19% mean	31±23% mean	n/a	48±24% mean	44±27% mean	18±13% mean	n/a

Conc, concentric phase; eccen, eccentric phase.

**Table 3:** Data description regarding sEMG activity in each study, in relation to exercise type and normalized sEMG activation expressed as % peak RMS.

Reference	Exercise	Gluteus maximus	Biceps Femoris	Vastus Lateralis	Rectus Femoris	Erector Spinae	Lumbar Multifidus
Lee et al. (2018) [27]	Deadlift	51.52 ± 6.0 peak RMS	57.45 ± 6.34% peak RMS	n/a	58.57 ± 13.73% peak RMS	n/a	n/a
	Romanian deadlift	46.88 ± 7.39% peak RMS	56.66 ± 18.56% peak RMS	n/a	25.26 ± 14.21% peak RMS	n/a	n/a
Snyder et al. (2017) [36]	Deadlift	~47% peak RMS	~28% peak RMS	~48% peak RMS	n/a	~73% peak RMS	n/a
	BallPro	~30% peak RMS	~25% peak RMS	~80% peak RMS	n/a	~53% peak RMS	n/a
	ToePro	~30% peak RMS	~31% peak RMS	~63% peak RMS	n/a	~58% peak RMS	n/a
Bezerra et al. (2013) [26]	Deadlift	n/a	100.1 ± 24.7% peak RMS	128.3 ± 33.9% peak RMS	n/a	n/a	112.7 ± 42.7% peak RMS
	Stiff leg deadlift	n/a	98.6 ± 28.5% peak RMS	101.1 ± 14.6% peak RMS	n/a	n/a	106 ± 20.5% peak RMS

BallPro, walk-in machine deadlift with feet ball-hand; RMS, root mean square; ToePro, walk-in machine deadlift with toes-hand.

**Table 4:** Data description regarding sEMG activity in each study, in relation to exercise type and normalized sEMG activation expressed as absolute RMS values in mV.

Reference	Exercise	Gluteus maximus	Biceps Femoris	Semitendinosus	Vastus Lateralis	Erector Spinae
Andersen et al. (2019) [8]	Deadlift	236 RMS (mV)	312 RMS (mV)	367 RMS (mV)	239 RMS (mV)	341 RMS (mV)
	DL FW-2EB	231 RMS (mV)	313 RMS (mV)	359 RMS (mV)	234 RMS (mV)	330 RMS (mV)
	DL FW-4EB	250 RMS (mV)	326 RMS (mV)	375 RMS (mV)	238 RMS (mV)	357 RMS (mV)
McAllister et al. (2014) [44]	Romanian deadlift	n/a	~360 RMS (mV) conc ~300 RMS (mV) eccen	~810 RMS (mV) conc ~790 RMS (mV) eccen	n/a	~210 RMS (mV) conc
	Glute ham-raise	n/a	~380 RMS (mV) conc ~160 RMS (mV) eccen	~1180 RMS (mV) conc ~490 RMS (mV) eccen	n/a	~430 RMS (mV) conc
	Good morning	n/a	~290 RMS (mV) conc ~210 RMS (mV) eccen	~910 RMS (mV) conc ~590 RMS (mV) eccen	n/a	~205 RMS (mV) conc
	Prone leg curl	n/a	~240 RMS (mV) conc ~85 RMS (mV) eccen	~870 RMS (mV) conc ~330 RMS (mV) eccen	n/a	~255 RMS (mV) conc

Conc, concentric phase; eccen, eccentric phase; EB, elastic bands; FW, free weight; mV, microvolts; RMS, root mean square.

**Table 5:** Data description regarding muscle activation in mV expressed as a percentage of EMG (mV) during 1RM effort.

Reference	Exercise	Gluteus maximus	Biceps Femoris	Vastus Lateralis	Vastus Medialis	Rectus Femoris	Erector Spinae
Korak et al. (2018) [39]	Deadlift	72% 1RM	82% 1RM	104% 1RM	92%1RM	105%1RM	n/a
	Parallel back squat	80% 1RM	78% 1RM	97% 1RM	96%1RM	102%1RM	n/a
	Parallel front squat	94% 1RM	81% 1RM	102% 1RM	98%1RM	101%1RM	n/a
Nijem et al. (2016) [37]	Deadlift	82.5 ± 6.9% 1RM	n/a	115.9 ± 30.1% 1RM	n/a	n/a	97.9 ± 8.7% 1RM
	Deadlift with chains	76.8 ± 6.8% 1RM	n/a	123.3 ± 45.1% 1RM	n/a	n/a	93.2 ± 11% 1RM
Camara et al. (2016) [38]	Deadlift	n/a	83.5 ± 19%1RM conc 34.7 ± 11%1RM eccen	96.8 ± 22%1RM conc 55.9 ± 12.6%1RM eccen	n/a	n/a	98.9 ± 26% 1RM conc 75.3 ± 28% 1RM eccen
	Hexagonal bar deadlift	n/a	72.3 ± 20%1RM conc 31.5 ± 10%1RM eccen	119.9 ± 22%1RM conc 87.9 ± 31%1RM eccen	n/a	n/a	88 ± 27% 1RM conc 61.4 ± 21% 1RM eccen

1RM, 1 repetition maximum; concentric phase; eccen, eccentric phase; RMS, root mean square.

Most researched *Deadlift* variants include the *Conventional Barbell Deadlift* (10/19 studies) [8, 13, 22, 27, 28, 32, 36–39] and the *Stiff Leg Deadlift* (6/19 studies) [16, 35, 40–43], which are followed by *Unilateral Stiff Leg Deadlift* (2/19 studies) [16, 43], *Romanian Deadlift* (2/19 studies) [27, 44] and *Hexagonal Bar Deadlift* (2/19 studies) [22, 38] (Table 1).

It is also important to clarify that exercises such as “*Olympic Barbell Deadlift*”, “*Straight Bar Deadlift”*, *“Barbell Deadlift”*, *“No Chains Deadlift*” and “*Conventional Barbell Deadlift*” all refer to the same exercise, so “*Deadlift*” will be used for all cases.

### Concentric and eccentric phases

Generally, studies analyzing electromyographical data assess muscle activation on each repetition, treating it as a single unit. Nonetheless, it has been reported that electromyographical activity could differ significantly between concentric and eccentric phases of the movement. Therefore, some authors have already carried out this division in their research [45–47]. Not all studies included in the current review divided sEMG exercises into concentric and eccentric phases. In fact, only seven studies performed such a subdivision [16, 28, 34, 37, 38, 40, 41], in which the concentric phase showed greater muscle activation than the eccentric phase for every single case.

### Muscle activation

The biceps femoris has been the most investigated muscle in terms of sEMG for the *Deadlift* exercise and its variants (13/19 studies). Gluteus maximus is the next muscle most evaluated (10/19) followed by vastus lateralis and erector spinae muscles (9/19). The semitendinosus and rectus femoris are positioned in fourth position (5/19) followed by vastus medialis, external oblique and medial gastrocnemius (3/19) (Table 1).

Due to the diversity regarding methodology, it was considered appropriate to report the results by grouping the studies according to the sEMG normalization process carried out in each study (mean or peak % MVIC, % peak RMS, RMS mV or % 1RM).

Studies in which muscle activation was expressed as a mean or peak % MVIC are shown in Table 2. Erector spinae showed the greatest muscle activation during the *Stiff Leg Deadlift* exercise [41], and also showed a similar muscle activation than the gluteus maximus or biceps femoris during *Deadlift* and *Hexagonal Bar Deadlift* exercises [22]. Except for the *Deadlift* exercise [22], the gluteus maximus showed greater muscle activation than biceps femoris [13, 22, 40, 41]. When comparing muscle activation within the hamstrings, there was a greater activation for the semitendinosus muscle than the biceps femoris during *Stiff Leg Deadlift* [16, 41, 42], which is even more pronounced when performing *Unilateral Stiff Leg Deadlift* [16]. The concentric phase showed a greater activation in the gluteus maximus and hamstring muscles than the eccentric phase for all exercises evaluated [16, 40, 41] (Table 2).

Data regarding muscle activation expressed as percentage peak RMS (% peak RMS) are shown in Table 3. The erector spinae and lumbar multifidus showed greater muscle activation than the gluteus maximus and biceps femoris [26, 36]. However, conflicting results have been reported for the *Deadlift* exercise. Lee et al. [27] reported more activation in the biceps femoris than the gluteus maximus, while Snyder et al. [36] reported more activation in the gluteus maximus than the biceps femoris (Table 3). Whereas the vastus lateralis showed greater muscle activation than the biceps femoris [26, 36], and the rectus femoris showed greater muscle activation than the biceps femoris and gluteus maximus during *Deadlift* exercise [27] (Table 3).

Data regarding muscle activation expressed as RMS in mV are shown in Table 4. Erector spinae and semitendinosus are the most activated muscle in the *Deadlift* exercise [22]. When comparing muscle activation within the hamstrings, there was a greater activation recorded for the semitendinosus muscle in comparison to that for the biceps femoris [22, 44] (Table 4). The concentric phase showed greater activation than the eccentric phase in all muscles and exercises evaluated [44].

Data regarding muscle activation in mV expressed as a percentage of sEMG (mV) during a 1RM effort are shown in Table 5. The Erector spinae presented higher muscle activation than the gluteus maximus [37] and biceps femoris [38]. The vastus lateralis and vastus medialis showed greater muscle activation than the biceps femoris and gluteus maximus during *Deadlift* exercises and its variants [37–39]. The concentric phase showed greater activation in the biceps femoris, vastus lateralis and erector spinae than the eccentric phase during the *Deadlift* exercise as well as during the hexagonal bar *Deadlift* exercise [38].

## Discussion

The main aim of the present study was to carry out a comprehensive literature review assessing muscle activation measured with sEMG when performing the *Deadlift* exercise and all its variants.

The most relevant results compiled from the literature review revealed that the biceps femoris is the most evaluated muscle when performing this kind of exercises [8, 13, 16, 22, 26, 27, 36–39, 41, 42, 44, 48], followed immediately by the gluteus maximus [8, 13, 22, 27, 36–41].

Erector spinae presented higher muscle activation than the gluteus maximus and the biceps femoris muscles for all exercises [8, 26, 37, 38, 41]. Only one study presented contrary outcomes, showing lower muscle activation in the erector spinae than the biceps femoris and semitendinosus during the *Romanian Deadlift* exercise [36].

Another important finding in the current review was that muscles from the quadriceps complex appeared to elicit the greatest muscle activation compared to the gluteus maximus and hamstrings muscles for *Deadlift* exercise [26, 27, 36–39]. Furthermore, the semitendinosus generally tended to elicit slightly greater muscle activation than the biceps femoris within the hamstring complex [8, 44].

### Methodological issues

One concern about the findings of the review was the lack of unification of collecting data methodological process amongst studies. This includes the kind of muscle contraction evaluated, the number of participants, the participants’ resistance training experience, exercise intensity during evaluation, sEMG collection method, electrode location, and number of evaluation days. All studies following a specific methodology process had diverse aims and different outcomes, which made difficult to deliver consistent results. Only one study evaluated just an isometric position of the movement, the preparatory position [35], whereas the rest evaluated exercises from a dynamic perspective.

The included studies were variate in number of participants (8–34) but similar in their sample population ages (18–34), who had a minimum of 6 months resistance training experience. It is important to highlight the impact that training status have upon muscle activation pattern, since familiarization with the movement could substantially modify muscle activation elicited during each exercise [49–51]. Furthermore, twelve of the studies had a male sample, while the rest combined both genders [34–36, 41, 43], and only two studies included exclusively females [39, 40]. This raises the necessity to invest more research into females in this field.

In line with previous reviews, exercise loading for sEMG recordings has been one of the biggest concerns [52–54]. Only two studies performed same 1RM intensity [14, 25], whereas others performed exercises at a predetermined repetition maximum load, and the rest measured a number of repetitions within a range of 65–85% 1RM (Table 1). Differences in the applied methodology should be reduced for future studies, providing an enhanced outcomes reliability [42].

No unified criterion has been followed in categories of time management during exercise phase among study methodologies, which could also be treated as a potential bias risk. For future studies focused on sEMG, it would be of significant interest to report divided electromyographical data into concentric and eccentric phases, as well as exercise timing. Such information would help coaches and trainers when choosing one or another exercise for a concrete target when prescribing an optimized training [7].

In relation to the electrode location, reports on surface recording of sEMG should include electrode shape and size, interelectrode distance, electrode location and orientation over muscle with respect to tendons and fiber direction among others (Merletti & Di Torino, 1999). It is vital to report in detail the placement of electrodes over the muscle belly when we aim to compare outcomes with other similar studies.

Different protocols for surface electromyography electrode placement have been described in the literature. One of the most popular protocols is the SENIAM Guidelines (Surface ElectroMyoGraphy for the Non-Invasive Assessment of Muscles). Eight studies following the SENIAM Guidelines have been included in our review [8, 16, 22, 35–37, 41, 42]. The rest followed some other Guidelines or a previous reference, and only four studies did not report any protocol for electrode location [26, 28, 32, 34].

In regard to interelectrode distance, five studies reported using the recommended 2 cm center-to-center distance between electrodes according to SENIAM Guidelines [8, 22, 27, 35, 43]. In addition to not following these Guidelines, some other studies also did not report the inter-electrode distance [26, 32, 34, 38–40, 44]. Furthermore, four studies reported to have placed the electrodes with a center-to-center distance ranging between 15–35mm but different from 20 mm [13, 16, 28, 37, 41]. The higher the interelectrode distance, the wider the detection volume and consequently the detected amplitude [55]. Future research should attempt to follow established Guidelines, so they can reach optimum research quality and diminish the risk of data collection bias.

On the other hand, most of the reviewed studies included between 2–4 days/sessions (visits to the laboratory) for the measurement process, normally leaving 2–7 days’ rest between each visit. Tasks performed during those days cover anthropometric data gathering, familiarization with exercises, RM testing and sEMG data collection. To ensure reliable sEMG data outcomes, sEMG data must be collected at the same session [8]. Otherwise, some studies collected sEMG data on two different days, which might have entailed electrode location mistakes [32, 40, 41, 44].

In order to avoid fatigue bias risks, a randomized counterbalanced order for exercise testing was followed in all studies but one, which followed a preset exercise order [41]. In addition with the same aim, a minimum break of 2–5 min was considered between exercise testing trials [22, 27, 28, 34, 36, 37, 40–42, 44].

Most studies did not report hand grip and stance position in any depth of detail. Some studies allowed a preferred stance position for each participant but maintained the same for all exercises tested [8, 22], whereas others also indicated a hand grip slightly wider than shoulder width [26, 27, 32, 40–43].

Likewise, information about MVIC should be strictly reported. Our reviewed studies reported a range between 2–3 trials, 3–5 seconds holding and 15–60 seconds rest between trials [13, 16, 22, 32, 40, 43].

### Other *Deadlift* variations

Apart from the above-mentioned *Deadlift* exercises, there are some other studies which focused on less conventional variants of this movement. The *Good Morning* exercise appears to be an appropriate substitute to *Romanian Deadlift* when it is preferable to place the load on the back instead of lifting it from the floor. *Good Morning* provokes a similar muscular pattern activation as *Romanian Deadlift*, but it showed more muscle activation for the semitendinosus and less muscle activation for the biceps femoris than *Romanian Deadlift* [44].

In addition, some authors proposed interesting alternatives for the *Deadlift* exercise with the goal of overcoming the sticking region. This involves a phase during the lift in which there is a mechanical disadvantage that elevates injury risk and leads to a deceleration on the speed lift [56]. In relation to this issue, Nijem et al. (2016) compared *Deadlift* versus *Deadlift with chains* and reported the existence of a lightest load at the sticking point which would allow one to maintain a neutral spine during *Deadlift with chains*. Regarding muscle activation, there were significant differences for the gluteus maximus muscle, which present greater activity during *Deadlift* than *Deadlift with chains*. Furthermore, Andersen et al. (2019) reported another resource by using the addition of elastic bands attached to the ceiling to displace the sticking point. This method would reduce the load from lower phases of the lift and increase the resistance as the bar goes up.

Elastic bands have also been used as a tool in *Deadlift* learning processes, when the athlete is not ready to lift high loads with a proper technique or in those cases when some injury prevents the athlete from using conventional resistance equipment. Muscular activation presented during elastic bands *Stiff Leg Deadlift* was lower than that elicited during free weights *Stiff Leg Deadlift*, with significant differences when referring to the gluteus maximus, biceps femoris and semitendinosus muscles [41].

Furthermore, it should be noted that if your aim is to increase muscle activation from forearm musculature during the *Deadlift* exercise, it is recommended to use a Fat Gripz device, a wider grip implement that sticks to the bar. Worth mentioning that a significant reduction in 1RM strength would appear when using this kind of implement [32].

### Comparing *Deadlift* to other exercises

Some studies included in this review also compared muscle activation elicited during *Deadlift* exercises versus other typical weight bearing exercises performed in weight rooms. McCurdy et al. (2018) reported significantly greater muscle activation for the gluteus maximus and hamstring muscles during *Modified Single Leg Squat* in comparison to *Back Squat* and *Stiff Leg Deadlift*. Whereas, Korak et al. (2018) reported the highest muscle activation for the gluteus maximus during *Front Squat* comparing to *Deadlift* exercise, with no differences for this muscle between *Front* and *Back Squat*.

Moreover, the *Hip Thrust* exercise has also been found to elicit greater muscle activation for the gluteus maximus than *Deadlift* and *Hexagonal Bar Deadlift*. Also, lower muscle activation for the biceps femoris muscle was shown during *Hip Thrust* compared to *Deadlift*. No muscle activation differences were presented among those three exercises for the erector spinae muscle. Hence, a greater torque and greater stress in the hip joint during *Deadlift* compared to both other exercises was also reported [22].

Additionally, several authors have compared *Deadlift* exercises to single joint and machine-based exercises in their research. For example, Bourne et al. (2017) reported significantly greater muscle activation during *45º Hip Extension* and *Nordic Hamstring Exercise* than *Stiff Leg Deadlift* and *Unilateral Stiff Leg Deadlift* for biceps femoris and semitendinosus muscles. Similar results support these findings, showing a greater muscle activation during the *Nordic Hamstring Exercise* and during *Seated Leg Curl* for hamstring muscles in comparison to the muscle activation elicited for hamstring muscles during *Stiff Leg Deadlift* and *Unilateral Stiff Leg Deadlift* [43].

On the other hand, the *Prone Leg Curl* in machine was found to elicit higher muscle activation for both upper and lower sections of the biceps femoris muscle than during *Stiff Leg Deadlift* but showed no significant differences for the semitendinosus muscle (Schoenfeld et al., 2015). On the contrary, McAllister et al. (2014) reported greater biceps femoris muscle activation during *Romanian Deadlift* than during the *Prone Leg Curl*. It would be necessary to unify the muscle activation normalization method and protocol carried out. Likewise, researchers should ascertain a proportional exercise load when comparing bilateral multi joint exercises to single leg and machine-based exercises, in order to obtain consistent outcomes.

## Conclusions

After performing the current systematic and comprehensive review, several conclusions have been reached. Main findings outlined that:

1. Biceps femoris is the most studied muscle (13/19), followed by gluteus maximus (10/19), vastus lateralis and erector spinae (9/19) during *Deadlift* exercises.
2. Erector spinae and quadriceps muscles are more activated than gluteus maximus and biceps femoris muscles within *Deadlift* exercises (9/19).
3. Within the hamstring muscles complex, semitendinosus elicits slightly greater muscle activation than biceps femoris during *Deadlift* exercises (6/19).

Some recommendations for future research involving surface electromyography recordings are:

1. Participants training status and participants resistance training experience should be outlined in detail. Only 11/19 studies showed this information.
2. Exercise load quantification method during sEMG recordings must be standardized, so exercises could be comparable among them.
3. Taking into consideration the different muscle activation pattern reported during concentric and eccentric exercises phases, it is highly recommended to perform such subdivision for future studies.
4. A unified criterion upon methodology protocol is necessary in order to avoid several bias risks and report reliable outcomes when using surface electromyography recordings. Information regarding electrode location, number of testing days and sEMG normalization method should be strictly reported.

## Practical applications

Currently, *Deadlift* is an exercise frequently performed to improve the lower limb muscles, mainly biceps femoris and semitendinosus (hamstrings), and gluteus maximus. Based on this systematic review about the sEMG activity in the *Deadlift* exercise and its variants, it has been demonstrated that other muscles such as erector spinae and quadriceps are more activated than hamstrings and gluteus maximus, although some studies found conflicting results.

*Deadlift* exercise comprises a movement which could have a transference into daily life activities; also considered as one of the greatest compound lifts, as it involves several muscles groups coordination. A broad spectrum of *Deadlift* variants has been reported, so diverse applications for these exercises could merge, covering health, rehabilitation and performance environments.

Therefore, it must be considered that muscle activation would depend on the *Deadlift* variant performed. For instances, posterior thigh muscles would show greater muscle activity when performing exercises that holds the knees on a fixed and extended position (e.g. *Romanian Deadlift* or *Straight Leg Deadlift*). On the contrary, whether your goal is to maximize anterior thigh and lower back muscle activity, *Deadlift* would be the exercise of choice. *Hexagonal Bar Deadlift* also elicits a great anterior thigh muscle activity, but with a reduction on erector spinae muscle activity, turning this exercise into an appropriate *Deadlift* variant when athletes have lower back issues.

Hence, coaches, athletes and regular population ought to contemplate these findings when selecting the *Deadlift* exercise and its variants for their training programs, considering the individual training goals.

## Supporting information

S1 Checklist (DOCX)

S1 File (PDF)

## Abbreviations

*Exercises abbreviations*: BallPro: walk-in machine deadlift with feet ball-hand
DL: deadlift
EB: elastic bands
FW: free weights
ToePro: walk-in machine deadlift with toes-hand
1RM: 1 repetition maximum
*Other abbreviations*: Conc: concentric phase
eccen: eccentric phase
ISOpull: isometric pulls
mV: microvolts
RM: repetition maximum
RMS: root mean square
ROM: range of motion
sEMG: surface electromyography
