# Supplementary material for: Electromyographic activity in deadlift exercise and its variants. A systematic review
Source: PLoS One. 2020 Feb 27;15(2):e0229507. doi: 10.1371/journal.pone.0229507 (PMC7046193; doi:10.1371/journal.pone.0229507)
Supplement: S1 File — (PDF) [file pone.0229507.s002.pdf]

Electromyographic activity in deadlift exercise and its variants. A systematic review

Isabel Martín Fuentes, José María Muyor Rodríguez

Citation

Isabel Martín Fuentes, José María Muyor Rodríguez. Electromyographic activity in deadlift exercise and its variants. A systematic review. PROSPERO 2019 CRD42019138026 Available from:
https://www.crd.york.ac.uk/prospero/display_record.php?ID=CRD42019138026

Review question

How does muscles from both lower and upper limbs get involved in terms of neuromuscular excitation when performing exercises as Deadlift or any of its derivatives?

Searches

A literature search of PubMed, OVID, Scopus & Web of Science electronic databases was performed during March-April 2019.

Most articles found were written in English, but there were no language restrictions. Reviews, congress publications, theses, books, books chapters, abstracts, and studies with poor protocol description or insufficient data were not included. Studies whose participants did not met 6 months experience in resistance training were excluded. We also excluded all those in which participants were under 18 years old (due to underdevelopment of strength and coordination). Studies reporting only muscle activation of the upper limbs during Deadlift exercise were also considered.

Types of study to be included

No study restriction

Condition or domain being studied

Neuromuscular excitation from lower and upper limb muscles when performing Deadlift exercise and its derivatives was studied.

Deadlift exercise is a trend exercise performed in weight rooms. It involves several muscle groups from the whole body and it is considered to be an intelligent choice when strength and coordination developing is one of your targets.

In this line, appears to be of great importance to be acquainted of which neuromuscular excitation pattern elicit any different variation form of this exercise. In categories of exercise prescription for rehabilitation, return to play, or preventive injuries processes, it could be really advantageous to spread this kind of knowledge to all population.

Participants/population

Included healthy and trained participants (studied which sample size are under 18 years old athletes were excluded), with no injury issues at least during 6 previous months before measurements

Intervention(s), exposure(s)

(i) cross-sectional or longitudinal (experimental or cohorts) study design;

(ii) evaluated neuromuscular limb excitation during Deadlift exercise or derivatives;

(iii) included healthy and trained participants, with no injury issues at least during 6 previous months before measurements;

(iv) analyzed sEMG amplitude with surface electromyography devices (sEMG)

Comparator(s)/control
Not applicable

Context

Main outcome(s)
Differences in neuromuscular excitation between different variations of deadlift exercise

Timing and effect measures

Not applicable

Additional outcome(s)
None

Timing and effect measures

Not applicable

Data extraction (selection and coding)

Articles were selected by two independent reviewers (IMF, JMM) according to inclusion and exclusion criteria. After eliminating duplicates, titles and abstracts were analyzed. If there were not enough information, full text was obtained for evaluation. All studies identified from the database searching were downloaded into the software EndNote version X9 (Clarivate Analytics, New York, NY, USA). Every decision was approved by both reviewers, in case of disagreement a third reviewer was consulted (JMO). The whole searching process took 2 weeks.

During extraction data process the following information was collected from every single study: reference, exercises-movements measured, sample size (n), gender, age (years), experience (years), evaluated muscles, electrodes location, limb tested (n/dominant), EMG collection method, normalized EMG method, outcomes percentage maximal voluntary isometric contraction (% MVIC), main findings.

Muscle excitation was the main data gathered. All studies finally selected reported muscle excitation of every muscle and exercise separately.

Risk of bias (quality) assessment
The two independent review authors will independently assess the risk of bias in included studies by considering PEDro assessment quality scale

Strategy for data synthesis

All articles presented cross-sectional design. Data collected in this review could not be analyzed as a meta-analysis since there was not enough homogeneity in terms of the type of analysis and methods carried out in each study. Therefore, a qualitative review of the results was conducted.

As we did not find an unified criteria for EMG normalization method, studies founded were divided into different groups; data description regarding muscle excitation in relation to exercise type and normalized sEMG activity as a %MVIC, data expressed as absolute RMS values in mV, and data as a percentage of 1 repetition maximum.

Thus, results were discussed in relation to EMG normalization method, in order to conclude with reliable outcomes.

Analysis of subgroups or subsets
Not applicable

Contact details for further information
Isabel Martín Fuentes
imf902@ual.es

Organisational affiliation of the review
Universidad de Almería
https://www.ual.es/

Review team members and their organisational affiliations
Miss Isabel Martín Fuentes. Universidad de Almería
Dr José María Muyor Rodríguez. Universidad de Almería

Type and method of review
Narrative synthesis, Systematic review

Anticipated or actual start date
05 March 2019

Anticipated completion date
31 July 2019

Funding sources/sponsors
Not applicable

Conflicts of interest

Language
English

Country
Spain

Stage of review
Review Ongoing

Subject index terms status
Subject indexing assigned by CRD

Subject index terms
Exercise; Humans; Weight Lifting

Date of registration in PROSPERO
01 November 2019

Date of publication of this version
01 November 2019

Details of any existing review of the same topic by the same authors

Stage of review at time of this submission

Stage	Started	Completed
Preliminary searches	Yes	Yes
Piloting of the study selection process	Yes	Yes
Formal screening of search results against eligibility criteria	Yes	Yes
Data extraction	No	No
Risk of bias (quality) assessment	No	No
Data analysis	No	No

Versions

01 November 2019

PROSPERO
This information has been provided by the named contact for this review. CRD has accepted this information in good faith and registered the review in PROSPERO. The registrant confirms that the information supplied for this submission is accurate and complete. CRD bears no responsibility or liability for the content of this registration record, any associated files or external websites.
